# Association between dental diseases and history of stroke in the United States

**DOI:** 10.1002/cre2.416

**Published:** 2021-04-02

**Authors:** Hatem A. Alhadainy, Thomas Keefe, Amany H. Abdel‐Karim, Saleem Abdulrab, Esam Halboub

**Affiliations:** ^1^ Department of Endodontics, College of Dentistry Tanta University Tanta Egypt; ^2^ Department of Environmental and Radiological Health Sciences Colorado State University Fort Collins Colorado USA; ^3^ Department of Statistics, Mathematics and Insurance, Faculty of Commerce Tanta University Tanta Egypt; ^4^ Madinat Khalifa Health Centre Primary Health Care Corporation Doha Qatar; ^5^ Department of Maxillofacial Surgery and Diagnostic Sciences College of Dentistry, Jazan University Jazan Saudi Arabia; ^6^ Department of Oral Medicine, Oral Pathology and Oral Radiology Faculty of Dentistry, Sana'a University Sana'a Yemen

**Keywords:** association, dental diseases, NHANES, periodontal, stroke

## Abstract

**Objective:**

This study aimed to determine the potential association between the dental diseases and self‐reported history of stroke in the United States based on data from the Third National Health and Nutrition Examination Survey (NHANES III).

**Methods:**

Data were extracted from NHANES III. Dental variables were carious tooth surfaces, number of missing teeth, gingival bleeding, and periodontal pockets. Multiple logistic regression modeling was used to estimate the effect of these dental diseases on the self‐reported history of stroke with intent to adjust for the other potential determinants: age, sex, race, marital status, health insurance, education, exercise, body mass index, smoking, alcohol, hypertension, high serum cholesterol, and diabetes.

**Results:**

Number of missing teeth was found to be significantly associated with the self‐reported history of stroke. Associations between the self‐reported history of stroke and caries, gingival bleeding, or periodontal pockets were not statistically significant.

**Conclusions:**

Number of missing teeth was an independent determinant of the self‐reported history of stroke.

## 1 INTRODUCTION

The effects of poor dental health on systemic diseases are well‐documented, a matter that has led to a renewed interest in contribution of dental diseases to the systemic health outcomes. A report of the Surgeon General issued by United States Department of Health and Human Services (DHHS) about “Oral Health in America” identified the essential role that dental health plays in overall health and wellbeing. The report clearly indicated that the disparities in dental health status and access to dental care affected many of the United States people, and more specifically those with low income and members of racial/ethnic minority groups (National Institute of Dental and Craniofacial Research, 2000).

Research have focused on the potential impact of oral diseases on systemic health such as cardiovascular disease (CVD), diabetes, respiratory diseases and adverse pregnancy outcomes (Dietrich & Garcia, 2005). Seymour et al. (2007) stated that dental diseases may represent a risk factor for systemic diseases, and hence controlling dental diseases is an essential approach in the prevention and management of these systemic diseases.

According to the Global Burden of disease study, stroke was the second largest cause of death globally, resulting in an annual mean estimate of 5.5 million deaths (Johnson et al., 2019). Several epidemiologic studies have investigated the links between dental diseases and stroke. This link appears to be indirect or circumstantial, and the results from previous studies are inconsistent (Joshipura, 2002; Lin et al., 2019; Sim et al., 2008).

The type of association between dental diseases and stroke has not been established. Joshipura (2002) reported that out of nine studies that evaluated tooth loss and periodontitis as risk factors for stroke, seven studies showed significant associations. However, the results were not consistently replicated as other studies failed to report such association.

Several studies claimed periodontitis as a risk factor for cerebral stroke in adults (Abolfazli et al., 2011; Hashemipour et al., 2013; Kim et al., 2010; Lin et al., 2019; Sen et al., 2018; Sim et al., 2008), unlike Howell et al. (2001) and Scannapieco et al. (2003) who did not report such a link between periodontitis and stroke. Helfand et al. (2009) added that no firm conclusions could be drawn about differences in risk prediction among racial and ethnic groups. Amidst this controversy, a recent meta‐analysis study confirmed the association between periodontal diseases and stroke (Fagundes et al., 2019).

Tooth loss, the ultimate end‐result and remarkable indicator of periodontal disease, has been considered a risk factor for cerebral diseases and CVD (Lee et al., 2019), and recent systematic reviews confirmed this association between tooth loss and stroke in adult patients (Cheng et al., 2018; Fagundes et al., 2020). Otherwise, research on the association of dental diseases, other than periodontal diseases and tooth loss, with history of stroke has not yet established, and more studies are required to investigate this issue. Therefore, this cross‐sectional study aimed to determine the potential association between dental diseases and the self‐reported history of stroke in the United States.

## 2 MATERIALS AND METHODS

### 2.1 Study design

The study was of a cross‐section design that analyzed retrospective data extracted from the Third National Health and Nutrition Examination Survey (NHANES III) database.

### 2.2 Data

Adult and Examination Data Files from NHANES III were utilized in the current study. The Adult Data File contained data from the survey responses while the Examination Data File contained data from the dental examination. Basically, NHANES III has been planned, and is being conducted as a continuous annual survey. The continuous NHANES survey data were released on public use data files in 2‐year increments.

In preparing the data set for analysis for this study, both NHANES III data files were merged using the unique survey participant identification number to ensure that the appropriate information for each individual survey participant was linked. The NHANES III was designed as a nationwide probability sample and included a total number of 33,994 subjects. The dataset was extracted into a file using the standard variable names and labels as provided in the NHANES III data files. This research was carried out according to the guidelines of Colorado State University, Fort Collins, CO, and permission was granted to access the patients' data. Ethics Committee of Colorado State University approved the study protocol.

### 2.3 Study variables

#### 2.3.1 Dependent variable

*Dependent variable* was the self‐reported history of stroke, and was extracted from Adult Data File based on the participants' responses to the following question: “doctor ever told you had stroke?”

#### 2.3.2 Independent variables

1. *Dental variables* which were extracted from Examination File that contained data form dental examination. These variables included: (a) *Dental caries*: The total number of carious surfaces was calculated for each person and ranged from 0 to 114 surfaces. The number of carious surfaces was categorized into four groups: Group I: no caries on any surfaces (29.8%), Group II: 1–9 carious surfaces (20.3%), Group III: 10–28 carious surfaces (25.2%) and Group IV: 29–114 carious surfaces (24.7%). (b) *Missing teeth*: The total number of lost teeth was categorized into four groups: Group I: 0–3 missing teeth (30.7%), Group II: 4–10 (24.3%), Group III: 11–27 (23.5%) and Group IV: 28 missing teeth (21.5%). The edentulous patient was defined as the individual with 28 missing teeth regardless presence of the third molars and/or tooth agenesis. (c) *Periodontal attachment loss (PAL)*: PAL was evaluated using periodontal probe on teeth of randomly assigned half‐mouths; one upper quadrant or one lower quadrant was selected for this purpose. The buccal and mesial‐buccal aspects of each tooth were scored separately for each periodontal measurement. Two measurements in mm were made at each site: the distance from the free gingival margin (FGM) to the cemento‐enamel junction (CEJ) and the distance from the FGM to the bottom of gingival sulcus. PAL was calculated by subtracting the first measurement (distance from FGM to CEJ) from the second measurement (distance from the FGM to the base of the sulcus). PAL were recorded for each selected site of every examined tooth and scored for each person according to the total number and depth of measurement. Each examined tooth was scored as 0 for no PAL, 1 for each 1‐2 mm PAL, and 2 for each PAL more than 2 mm depth. The maximum score for a single tooth was 4 (for a tooth with two measurements ≥2 mm each). These scores were summed for each subject to obtain a total PAL score that ranged from 0 to 28. This score was categorized into four groups: Group I: 0–8 (49.8%), Group II: 9–12 (21.61%), Group III: 13–16 (16.62%) and Group IV: 17 to 28 (11.96%). (d) *Gingival bleeding*: Data on gingival bleeding were recorded for each surface of the examined tooth using dental sickle probe. The total number was categorized into three groups: Group I: no single tooth with gingival bleeding (39.7%), Group II: 1–2 teeth with gingival bleeding (34.2%) and Group III: 3 or more teeth with gingival bleeding (26.1%).
2. *Demographic variables* which included age, sex, race, marital status, health insurance coverage and education of the participant at the time of the survey. *Age* was restricted to ≥45 years at baseline and categorized into four 10‐year intervals: 45–54 (23.1%), 55–64 (24.9%), 65–74 (26.2%) and 75 years or more (25.8%). *Sex* was recorded as male or female. *Race* was classified in NHANES III as white, African American, and others. In the current study, race was recategorized as white and nonwhite due to the small number of subjects in the “others” category. *Marital status* was categorized into two categories: “married” for spouse in household, spouse not in household and living as married, and “not married” for separated, divorced, widowed and never‐married. *Health insurance* included data on two separate dichotomous variables (yes/no): the first variable was for general health insurance subscription to a plan that covered any routine doctor's care and the second variable was for subscription to a plan that covered dental care. *Years of education* ranged from 0 to 17 years on the basis of the reported highest grade or years of regular school completed. This variable was categorized into three groups: 0–9 years (elementary and junior high school), 10–12 years (high school), and 13–17 years (college and post‐graduate education).
3. *Systemic risk factors* which included variables with dichotomous (“yes/no”) responses to: hypertension, serum cholesterol, diabetes, exercise, alcohol, and smoking. Body mass index (BMI) was quantitative variable that computed from body weight and standing height. Documentation of hypertension, serum cholesterol level and diabetes were based on the subjects' responses to questions: “doctor ever told you had high blood pressure? high cholesterol level? and sugar/diabetes?,” respectively. Exercise was recorded based on the subjects' responses to a questionnaire items regarding various physical activities such as walking, jogging, skiing, weight‐lifting, swimming and sports. Any “yes” answer for any physical activities was considered “yes” for the exercise variable; the participant had to answer “no” for all physical activities to be considered as “no” for exercise variable. Alcohol consumption was based on the subjects' responses to a question about drinking any alcoholic beverages currently. Smoking status was about all types of smoking and tobacco use. The use of any tobacco regardless its quantity or mean of admission in any time was defined as “yes,” and the answer had to be “no” for all various NHANES III questions regarding means of tobacco use to be counted as “no” in this study.

Means and *SDs* for numerical variables (dental caries, missing teeth, bleeding, PAL, age, and BMI) were computed before being categorized. In general, categorization was based on sample quartiles for quantitative variables and on combining any small‐frequency categories for qualitative variables.

### 2.4 Statistical methodology

Logistic regression was used in this study using Analytics Software & Solutions “SAS” package (SAS Institute, Cary, NC). Simple logistic regression (SLR) was used to evaluate the effect of each of the independent variables individually on stroke and variable was eliminated from further consideration if it was found to be nonsignificant at *p*‐value ≤ 0.25. Multiple logistic regression (MLR) was then conducted to evaluate the association between stroke and the independent variables that were significant in SLR analysis. Only independent variables with a *p*‐value ≤ 0.05 were considered significant in the final MLR model, and Odds ratio (OR) was computed for each independent variable.

## 3 RESULTS

### 3.1 Descriptive statistics

From the NHANES III main sample, after age restriction to ≥45 years, the resultant sample included in this study was 8634 subjects. The mean age was 65.5 ± 12.3 years and females represented more than half of the sample (52.3%). The majority of respondents were from white race since they represented 75% of the sample and married individuals were 61.76%. There was 91.4% of the study population covered for hospital expenses or routine doctor's care and only 41% covered for dental care. Regarding years of education, 37.9% completed 10–12 years and 22.3% completed 13–17 years of education. From the total sample, 531 subjects (6.15%) reported medical history of having a stroke.

The mean value of the number of carious tooth surfaces was 17.2 ± 14.3 and the mean value of the number of missing teeth was 12.1 ± 10.6. Only 4331 individuals were assessed for PAL; the mean of total score of PAL was 9.2 ± 5.7 mm. Gingival bleeding was examined in 4353 subjects; the mean value of this variable was 1.8 ± 1.3.

The mean BMI was 27.4 ± 5.5. From the study population, 45.7% reported practicing regularly at least one kind of physical exercises. Up to 55.4% of the sample reported consuming alcohol and 71.5% of them reported indulging in smoking and/or tobacco use. The self‐reported medical history revealed that 41.8% were hypertensive, 13.7% were diabetic and 39.4% had a high level of serum cholesterol.

### 3.2 Regression analysis

SLR for history of stroke with each independent variable indicated that general health insurance, dental insurance, marital status and BMI were nonsignificant (*p*‐value = 0.83, 0.76, 0.60, and 0.64, respectively). Regarding dental variables, PAL variable was nonsignificant in any of its categories (*p*‐value = 0.37, 0.74, and 0.65 for Group I, II, and III, respectively).

The significant demographic variables in the SLR analyses were age, sex, race, and education. The significant systemic variables were hypertension, serum cholesterol level, diabetes, exercise, alcohol, and smoking. Significant dental variables were dental caries, missing teeth, and gingival bleeding. Table 1 shows the *p*‐value and odds ratio (OR) along with 95% confidence intervals (95% CI) of the SLR for these variables.

**TABLE 1:** The significant independent variables in univariate logistic regression analyses

Variable	Crude OR	95% confidence intervals		*p*‐value
Caries				
1–9	1.53	0.74	2.57	0.71
10–28	2.03	0.88	2.70	0.55
29+	2.58	2.11	3.09	0.07
Missing teeth				
4–10	1.55	1.47	1.87	0.01
11–27	2.61	1.94	2.75	0.01
28	2.90	2.53	3.11	0.01
Gingival bleeding				
1	1.18	1.06	1.55	0.08
3+	1.68	0.74	1.83	0.71
Age				
45–54	0.42	0.28	0.67	0.01
55–64	0.66	0.50	0.74	0.02
65–74	0.81	0.43	0.88	0.01
Years of education				
0–9	0.73	0.52	0.85	0.007
10–12	0.88	0.68	0.93	0.29
Sex				
Male	1.07	1.27	1.85	0.03
Race				
White	1.20	1.07	1.58	0.038
Exercise				
Yes	0.67	0.55	0.74	0.01
Smoking				
Yes	1.15	1.02	1.83	0.19
Alcohol				
Yes	2.64	2.13	3.42	0.01
Cholesterol				
Yes	1.20	1.07	1.49	0.09
Diabetes				
Yes	2.37	1.93	2.71	0.01
Hypertension				
Yes	2.66	2.21	3.18	0.01

*Note*: Reference categories for independent variables are 0 for caries, 0–3 for missing teeth, 0 for gingival bleeding, 75+ for age, 13–17 for years of education, female for sex, nonwhite for race, no for exercise, no for smoking, no for alcohol, no for cholesterol, no for diabetes, and no for hypertension.

Significant variables in the SLR (*p* < 0.25) were evaluated simultaneously via MLR. The final model for MLR included only independent variables significant at *p* value ≤ 0.05. These variables were number of missing teeth, age, sex, exercise, alcohol, hypertension, and diabetes. The *p*‐values, OR and 95% CI of the significant variables of MLR model are summarized in Table 2.

**TABLE 2:** The significant independent variables included in the final multiple logistic regression model

Variable	Adjusted (OR)	95% confidence intervals		*p*‐value
Missing teeth				
4–10	1.72	1.36	2.29	0.016
11–27	2.40	2.24	2.69	0.02
28	3.09	2.97	3.12	0.01
Age				
45–54	0.46	0.09	0.74	0.01
55–64	0.60	0.39	0.86	0.03
65–74	0.72	0.45	0.92	0.05
Sex				
Male	1.40	1.11	1.65	0.02
Exercise				
Yes	0.76	0.57	0.81	0.01
Alcohol				
Yes	1.78	1.47	2.08	0.01
Hypertension				
Yes	3.02	2.69	3.35	0.01
Diabetes				
Yes	1.92	1.56	2.49	0.01

*Note*: Reference categories for independent variables are 0–3 for missing teeth, 75+ for age, female for sex, no for exercise, no for alcohol, no for hypertension, and no for diabetes.

The number of missing teeth was the only dental variable that remained significant in the final model. Compared to those who had 0–3 missing teeth, those who had 4–10 missing teeth showed OR of 1.72 (95% CI: 1.36–2.29) and those who had 11–27 missing teeth were found to be significantly more likely to report stroke with OR = 2.40 (95% CI: 2.24–2.69). The OR of 28 missing teeth and edentulous patients was the highest (3.09, 95% CI: 2.97–3.12).

Age and sex were the only demographic variables to be significant in the final MLR. Compared to those aged 75 years and older, the patients aged 45–54 years were significantly the least likely to report stroke with OR = 0.46 (95% CI: 0.29–0.74) followed by patients aged 55–64 years with OR = 0.6 (95% CI: 0.39–0.86). Meanwhile patients aged 65–74 years were insignificantly less likely to report stroke with OR = 0.72 (95% CI: 0.45–0.92). Reporting stroke by males was significantly more likely compared to females with OR = 1.4 (95% CI: 1.11–1.65), and significantly less likely by those who reported practicing any exercises compared to who did not (OR = 0.76, 95% CI: 0.57–0.81). Reporting stroke by hypertensive and diabetic patients and alcoholics was significantly more likely compared to their counterparts (OR = 3.02, 95% CI: 2.69–3.35 for hypertension; OR = 1.92, 95% CI: 1.56–2.49 for diabetes; and OR = 1.78, 95% CI: 1.47–2.08 for alcohol).

## 4 DISCUSSION

It has been suggested that tooth loss can be considered as a marker of periodontal disease and/or a history of damage of tooth structures. Therefore, it seems logic to associate between history of dental diseases that eventually ended with extraction and history of having stroke. However, tooth loss may also be a result of systemic disease such as diabetes. Lee et al. (2006) stated that it is unclear whether cumulative periodontal disease is an independent risk factor for stroke or a risk marker for the disease. Meurman and Hämäläinen (2006) concluded that the scientific evidence is still weak on these interactions.

In this study, the number of missing teeth was associated with the history of stroke, supporting the finding of Lee et al. (2019), Del Brutto et al. (2017), and Lafon et al. (2014). Although the study of Joshy et al. (2016) showed that tooth loss was not associated with stroke, a recent meta‐analysis study by Cheng et al. (2018), showed that the increase of one missing tooth was associated with a 1.5% increment in stroke risk. The results of this study suggested a significant association between stroke and missing teeth. Thus, the tooth loss found to be a significant risk factor for stroke.

Periodontitis is caused by local infections with periodontal pathogens, which in turn leads to systemic reactions, such as inflammation and immunological reactions (Tonomura et al., 2016). With an increase in local infection in the periodontal pockets, a systemic inflammatory response is triggered leading to the release of inflammatory mediators (Hosomi et al., 2012) such as fibrinogen and may increase platelet aggregation, thus contributing to atherosclerosis and thrombosis. The stages of the Periodontal profile class have been significantly associated with prevalent diabetes, coronary heart disease as well as other dental and medical outcomes including attachment loss, tooth loss, systemic biomarkers, and stroke (Beck et al., 2018). Tooth loss is the ultimate stage of periodontal disease and may be associated with an increase in C‐reactive protein (CRP), which itself is implicated in atherosclerosis and thus in the occurrence of stroke (You et al., 2009).

Neither gingival bleeding nor periodontal pockets/periodontal attachment loss were significant determinants of self‐reported stroke in our study. This finding agrees with other studies that did not conclude any association between periodontal diseases and stroke (Meurman & Hämäläinen, 2006; Scannapieco et al., 2003). Contradicting, Sim et al. (2008) found that periodontal diseases to be associated with nonfatal stroke in Korean adults.

Meurman et al. (2004) argued that due to the multifactorial nature of periodontal infection and CVD, confirming a causal association is difficult and hence the published results are conflicting. In addition, the inadequate control of numerous confounding factors resulted in an overestimation and imprecise measurement of the predictors, or over‐adjustment for confounders, resulting in underestimation of the risks.

The current study revealed that exercising decreases the chance of being stroked. Alcohol consumption was found to be a risk factor for stroke which agreed with the report of the American Heart Association (Benjamin et al., 2017). In addition, the current study found that individuals who suffered from hypertension and diabetes would be stroked more likely. These results agreed with Tun et al. (2017) who reported a significant association between stroke and diabetes. Diabetes plays a major role in lowering body defenses against infections (Meurman et al., 2004) which in turn play major roles in the progress of stroke. Diabetes also has a direct effect on the progress of dental disease, especially gingival inflammation and periodontitis (Taylor et al., 2004).

The most recent “Morbidity and mortality weekly report” (MMWR) published by Centre for Disease Control and Prevention (CDC) concluded that adults aged ≥50 years who had a dental exam as part of the NHANES and reported selected chronic conditions (stroke included) were significantly more likely to have severe tooth loss than those without chronic conditions (Parker et al., 2020).

NHANES III data were used to investigate the possible association between dental diseases and self‐reported history of stroke. However, limitations of these data included modifications in the questionnaires and examination components over the course of the survey, data were not available for certain variables for the full course of the study, and data on history of stroke were extracted from survey responses that are susceptible to information bias.

## 5 CONCLUSIONS

History of dental diseases, as indicated by the number of missing teeth, is associated with the history of stroke. Additional studies are recommended to determine the nature of this association and laboratory measures of infection and clinical evaluations should be used in longitudinal studies for further investigations.

## CONFLICT OF INTEREST

The authors confirm that they have no conflict of interest regarding this publication.

## AUTHOR CONTRIBUTIONS

Hatem A. Alhadainy and Thomas Keefe made conception and design of the study, acquisition and interpretation of data, and manuscript writing. Amany H. Abdel‐Karim contributed to statistical data analysis, interpretation of the outcomes, and critically revised the manuscript. Saleem Abdulrab and Esam Halboub contributed in critical review. All authors read and approved the final manuscript and agreed to be accountable for all aspects of the work.

## Data Availability

Data were extracted from Adult and Examination Data Files from the Third National Health and Nutrition Examination Survey (NHANES III), United States.
